# Supplementary material for: Long-term survival of patients receiving home hemodialysis with self-punctured arteriovenous access
Source: PLoS One. 2024 May 31;19(5):e0303055. doi: 10.1371/journal.pone.0303055 (PMC11142548; doi:10.1371/journal.pone.0303055)
Supplement: S1 File — (PDF) [file pone.0303055.s001.pdf]

| No | Female | Age at RRT Initiation | Age at HHD initiation | Cause of ESKD | Diabetes |
|---|---|---|---|---|---|
| 1 | 0 | 37 | 41 | Unknown | Unknown |
| 2 | 0 | 49 | 51 | HNS | 0 |
| 3 | 0 | 25 | 30 | CGN | 0 |
| 4 | 0 | 46 | 48 | DN | 1 |
| 5 | 0 | 46 | 56 | HNS | 0 |
| 6 | 0 | 31 | 53 | CGN | 0 |
| 7 | 0 | 40 | 51 | CGN | 0 |
| 8 | 0 | 42 | 48 | CGN | 0 |
| 9 | 0 | 55 | 56 | DN | 1 |
| 10 | 0 | 33 | 36 | HNS | 0 |
| 11 | 1 | 22 | 25 | Other | 0 |
| 12 | 0 | 19 | 27 | Unknown | 0 |
| 13 | 0 | 44 | 48 | DN | 1 |
| 14 | 0 | 40 | 41 | CGN | 0 |
| 15 | 0 | 49 | 49 | DN | 1 |
| 16 | 0 | 32 | 33 | CGN | 0 |
| 17 | 0 | 49 | 51 | CGN | 0 |
| 18 | 1 | 38 | 49 | CGN | 0 |
| 19 | 1 | 34 | 42 | Other | 0 |
| 20 | 0 | 38 | 40 | CGN | 0 |
| 21 | 1 | 36 | 42 | CGN | 0 |
| 22 | 1 | 35 | 36 | PKD | 0 |
| 23 | 0 | 46 | 47 | CGN | 0 |
| 24 | 0 | 50 | 58 | CGN | 0 |
| 25 | 0 | 57 | 59 | HNS | 0 |
| 26 | 1 | 42 | 56 | CGN | 0 |
| 27 | 1 | 47 | 52 | DN | 1 |
| 28 | 0 | 38 | 47 | Other | 0 |
| 29 | 0 | 39 | 43 | Other | 0 |
| 30 | 0 | 36 | 64 | CGN | 0 |
| 31 | 0 | 38 | 48 | Unknown | 0 |
| 32 | 0 | 65 | 69 | HNS | 0 |
| 33 | 0 | 56 | 57 | DN | 1 |
| 34 | 0 | 62 | 68 | CGN | 0 |
| 35 | 0 | 57 | 60 | NS | 0 |
| 36 | 0 | 54 | 55 | CGN | 1 |

| | | | | | |
|---|---|---|---|---|---|
| 37 | 0 | 53 | 53 | DN | 1 |
| 38 | 0 | 51 | 52 | HNS | 0 |
| 39 | 0 | 41 | 41 | PKD | 0 |
| 40 | 0 | 57 | 62 | CGN | 0 |
| 41 | 0 | 57 | 58 | CGN | 0 |
| 42 | 0 | 40 | 42 | DN | 1 |
| 43 | 1 | 43 | 44 | CGN | 0 |
| 44 | 0 | 44 | 45 | DN | 1 |
| 45 | 0 | 39 | 45 | NS | 0 |
| 46 | 0 | 65 | 66 | DN | 1 |
| 47 | 0 | 47 | 47 | HNS | 0 |
| 48 | 0 | 47 | 48 | CGN | 0 |
| 49 | 0 | 52 | 52 | CGN | 0 |
| 50 | 0 | 36 | 36 | CGN | 0 |
| 51 | 0 | 54 | 54 | Other | 0 |
| 52 | 0 | 45 | 45 | DN | 1 |
| 53 | 0 | 58 | 63 | NS | 0 |
| 54 | 0 | 48 | 48 | CGN | 0 |
| 55 | 0 | 62 | 62 | HNS | 0 |
| 56 | 0 | 50 | 52 | HNS | 0 |
| 57 | 0 | 50 | 50 | CGN | 1 |
| 58 | 0 | 50 | 53 | PKD | 0 |
| 59 | 0 | 45 | 46 | PKD | 0 |
| 60 | 0 | 59 | 60 | Unknown | 0 |
| 61 | 0 | 56 | 68 | CGN | 0 |
| 62 | 0 | 45 | 46 | HNS | 0 |
| 63 | 0 | 47 | 47 | DN | 1 |
| 64 | 0 | 57 | 58 | HNS | 1 |
| 65 | 0 | 61 | 62 | PKD | 0 |
| 66 | 0 | 47 | 48 | CGN | 0 |
| 67 | 0 | 42 | 43 | DN | 1 |
| 68 | 0 | 61 | 65 | CGN | 0 |
| 69 | 0 | 24 | 28 | NS | 0 |
| 70 | 1 | 49 | 63 | CGN | 0 |
| 71 | 0 | 60 | 63 | Unknown | 0 |
| 72 | 0 | 68 | 69 | NS | 0 |
| 73 | 1 | 48 | 48 | Unknown | 0 |

| | | | | | |
|---|---|---|---|---|---|
| 74 | 0 | 60 | 71 | DN | 1 |
| 75 | 0 | 33 | 33 | DN | 1 |
| 76 | 1 | 59 | 67 | CGN | 0 |
| 77 | 1 | 68 | 68 | CGN | 0 |

| Diabetic Retinopathy | Coronary Artery Disease | Modality before HHD | Alb | sCr |
|---|---|---|---|---|
| Unknown | Unknown | 1 | #DIV/0! | #DIV/0! |
| 0 | 0 | 1 | #DIV/0! | #DIV/0! |
| 0 | 0 | 2 | #DIV/0! | #DIV/0! |
| 1 | 0 | 1 | 4.20 | 10.89 |
| 0 | 0 | 1 | 4.28 | 12.58 |
| 0 | 1 | 1 | 3.88 | 13.53 |
| 0 | 0 | 1 | 4.25 | 14.47 |
| 0 | 0 | 2 | 2.58 | 10.18 |
| 0 | 0 | 1 | 3.83 | 7.03 |
| 0 | 0 | 4 | 4.20 | 14.25 |
| 0 | 0 | 4 | 3.98 | 10.79 |
| 0 | 1 | 1 | 4.43 | 16.34 |
| 1 | 0 | 4 | 4.23 | 11.25 |
| 0 | 0 | 1 | 3.88 | 10.98 |
| 0 | 0 | 0 | 4.00 | 9.17 |
| 0 | 0 | 1 | 4.40 | 13.30 |
| 0 | 0 | 1 | 3.90 | 14.97 |
| 0 | 0 | 4 | 3.60 | 13.66 |
| 0 | 0 | 4 | 3.53 | 9.37 |
| 0 | 0 | 1 | 3.98 | 12.80 |
| 0 | 0 | 3 | 4.23 | 12.13 |
| 0 | 0 | 0 | 4.33 | 5.80 |
| 0 | 0 | 0 | 3.24 | 11.00 |
| 0 | 0 | 4 | 3.33 | 15.14 |
| 0 | 0 | 1 | 3.75 | 13.34 |
| 0 | 0 | 1 | 3.93 | 11.54 |
| 1 | 0 | 4 | 3.77 | 8.74 |
| 0 | 0 | 4 | 3.05 | 15.25 |
| 0 | 0 | 1 | 3.70 | 9.37 |
| 0 | 0 | 1 | 3.38 | 9.17 |
| 0 | 0 | 4 | 3.56 | 13.37 |
| 0 | 0 | 2 | 3.93 | 13.73 |
| 1 | 0 | 0 | 3.30 | 9.86 |
| 0 | 0 | 4 | 3.10 | 10.31 |
| 0 | 0 | 1 | 3.16 | 13.84 |
| 0 | 0 | 0 | 2.92 | 7.97 |

| | | | | |
|---|---|---|---|---|
| 1 | 0 | 0 | 2.70 | 3.31 |
| 0 | 0 | 1 | 3.64 | 6.43 |
| 0 | 0 | 0 | 4.00 | 12.94 |
| 0 | 0 | 2 | 3.77 | 13.40 |
| 0 | 0 | 0 | 3.78 | 7.02 |
| 0 | 1 | 1 | 4.03 | 17.01 |
| 0 | 0 | 0 | 3.72 | 13.13 |
| 1 | 0 | 0 | 4.43 | 5.87 |
| 0 | 0 | 4 | 3.23 | 11.07 |
| 1 | 0 | 0 | 3.80 | 8.89 |
| 0 | 0 | 0 | 4.03 | 12.87 |
| 0 | 0 | 0 | 3.62 | 8.97 |
| 0 | 0 | 0 | 3.58 | 6.95 |
| 0 | 0 | 0 | 3.80 | 14.23 |
| 0 | 0 | 1 | 3.80 | 8.06 |
| 0 | 0 | 1 | 4.20 | 10.88 |
| 0 | 0 | 1 | 3.37 | 13.33 |
| 0 | 0 | 1 | 4.42 | 10.44 |
| 0 | 0 | 0 | 3.94 | 12.89 |
| 0 | 0 | 4 | 3.92 | 15.98 |
| 0 | 0 | 0 | 3.65 | 7.55 |
| 0 | 0 | 1 | 3.67 | 13.58 |
| 0 | 0 | 1 | 4.33 | 10.95 |
| 0 | 0 | 0 | 3.90 | 15.55 |
| 0 | 0 | 4 | 3.05 | 11.63 |
| 0 | 0 | 0 | 4.30 | 12.60 |
| 0 | 0 | 0 | 3.53 | 11.53 |
| 0 | 0 | 0 | 3.97 | 10.40 |
| 0 | 0 | 0 | 3.35 | 8.35 |
| 0 | 0 | 0 | 4.50 | 16.21 |
| 0 | 0 | 0 | 3.08 | 6.49 |
| 0 | 0 | 4 | 3.02 | 10.51 |
| 0 | 0 | 1 | 4.28 | 16.78 |
| 0 | 0 | 1 | 3.97 | 12.24 |
| 0 | 0 | 4 | 3.70 | 12.82 |
| 0 | 0 | 1 | 3.82 | 10.27 |
| 0 | 0 | 0 | 4.22 | 7.33 |

| | | | | |
|---|---|---|---|---|
| 0 | 1 | 1 | 4.03 | 11.06 |
| 1 | 0 | 0 | 2.13 | 7.58 |
| 0 | 0 | 4 | 4.05 | 12.74 |
| 0 | 0 | 1 | 4.00 | 4.00 |

| UN | sK | cCa | P | PTH | β2MG | CRP | LDL-C | Hb |
|---|---|---|---|---|---|---|---|---|
| #DIV/0! | #DIV/0! | #DIV/0! | #DIV/0! | #DIV/0! | #DIV/0! | #DIV/0! | #DIV/0! | #DIV/0! |
| #DIV/0! | #DIV/0! | #DIV/0! | #DIV/0! | #DIV/0! | #DIV/0! | #DIV/0! | #DIV/0! | #DIV/0! |
| #DIV/0! | #DIV/0! | #DIV/0! | #DIV/0! | #DIV/0! | #DIV/0! | #DIV/0! | #DIV/0! | #DIV/0! |
| 52.00 | 4.2 | 8.0 | 2.5 | #DIV/0! | 25.9 | 0.10 | #DIV/0! | 10.2 |
| 59.80 | 6.4 | 8.9 | 5.1 | 533.3 | 22.5 | 0.17 | #DIV/0! | 10.3 |
| 63.40 | 5.2 | 10.4 | 5.1 | 407.5 | 25.8 | 0.18 | #DIV/0! | 9.5 |
| 80.50 | 6.2 | 9.0 | 6.3 | 498.0 | 29.2 | 0.52 | #DIV/0! | 8.3 |
| 37.33 | 4.6 | 10.5 | 5.7 | 97.0 | #DIV/0! | 1.04 | #DIV/0! | 9.7 |
| 61.67 | 4.7 | 8.4 | 5.0 | 300.0 | 32.2 | 0.10 | 72 | 9.1 |
| 44.00 | 3.9 | 10.0 | 7.0 | 190.0 | 41.3 | 0.86 | 92 | 13.1 |
| 50.00 | 5.2 | 8.7 | 8.9 | 389.0 | #DIV/0! | 0.10 | #DIV/0! | 10.7 |
| 75.83 | 5.8 | 7.7 | 8.4 | 22.2 | 26.7 | 0.10 | #DIV/0! | 9.8 |
| 72.00 | 5.2 | 6.8 | 6.1 | 245.5 | 36.3 | 0.28 | #DIV/0! | 9.8 |
| 72.67 | 3.9 | 8.6 | 5.3 | 405.5 | 23.0 | 0.36 | #DIV/0! | 10.4 |
| 62.17 | 5.2 | 8.7 | 4.7 | 150.5 | 21.7 | 0.11 | 37 | 9.8 |
| 54.67 | 4.2 | 9.2 | 3.8 | 224.0 | 18.1 | 0.10 | #DIV/0! | 10.1 |
| 74.67 | 5.1 | 8.8 | 5.2 | 272.3 | 30.3 | 0.10 | #DIV/0! | 9.2 |
| 69.83 | 5.0 | 8.2 | 4.7 | 62.0 | 33.8 | 0.11 | #DIV/0! | 11.2 |
| 56.17 | 5.4 | 9.3 | 6.4 | 116.3 | 22.0 | 0.11 | #DIV/0! | 12.0 |
| 83.50 | 5.8 | 6.7 | 6.2 | 367.7 | 32.4 | 0.18 | #DIV/0! | 9.5 |
| 51.00 | 4.9 | 9.1 | 3.5 | 431.0 | 30.1 | 0.10 | #DIV/0! | 9.4 |
| 55.83 | 4.3 | 8.7 | 5.2 | 539.3 | 11.5 | 0.31 | 130 | 10.8 |
| 52.20 | 5.0 | 9.3 | 5.7 | 261.0 | 23.0 | 0.10 | 111 | 9.4 |
| 65.67 | 4.9 | 9.3 | 5.3 | 56.0 | 30.2 | 0.10 | 96 | 11.9 |
| 62.75 | 4.7 | 8.8 | 6.9 | 126.0 | 34.9 | 0.86 | #DIV/0! | 10.7 |
| 67.00 | 5.2 | 9.6 | 5.1 | 101.4 | 36.7 | 0.10 | #DIV/0! | 10.2 |
| 58.00 | 6.1 | 10.1 | 6.7 | 49.0 | 31.1 | 0.10 | 97 | 10.4 |
| 89.33 | 6.4 | 9.8 | 8.0 | 28.0 | 30.3 | 0.20 | 69 | 10.6 |
| 48.00 | 6.1 | 9.5 | 3.4 | 111.5 | 27.2 | 0.24 | #DIV/0! | 12.7 |
| 37.17 | 4.5 | 10.3 | 3.8 | 203.8 | 36.8 | 0.11 | #DIV/0! | 10.5 |
| 43.60 | 5.6 | 9.4 | 6.5 | 415.0 | 28.7 | 0.10 | #DIV/0! | 10.7 |
| 59.00 | 5.3 | 8.8 | 6.0 | 86.1 | 32.2 | 0.19 | #DIV/0! | 10.5 |
| 60.00 | 5.1 | 7.8 | 5.6 | 281.1 | 18.2 | 0.13 | 82 | 10.4 |
| 71.00 | 4.8 | 9.2 | 5.6 | 183.2 | 38.0 | 0.10 | #DIV/0! | 10.8 |
| 79.20 | 5.5 | 10.1 | 6.4 | 243.9 | 32.0 | 1.62 | #DIV/0! | 10.6 |
| 56.00 | 4.3 | 9.0 | 5.4 | 272.9 | 21.6 | 0.36 | 110 | 10.7 |

| | | | | | | | | |
|---|---|---|---|---|---|---|---|---|
| 41.67 | 4.4 | 9.6 | 4.7 | 138.1 | #DIV/0! | 0.10 | 64 | 10.4 |
| 59.33 | 5.0 | 8.6 | 4.6 | 22.6 | 14.9 | 0.10 | 119 | 9.6 |
| 57.50 | 3.6 | 9.2 | 4.5 | 78.5 | 19.8 | 0.11 | 54 | 8.9 |
| 57.17 | 5.2 | 9.7 | 5.6 | 226.5 | 27.4 | 0.10 | #DIV/0! | 11.8 |
| 89.67 | 5.6 | 8.5 | 5.0 | 270.9 | 17.7 | 0.10 | 73 | 9.8 |
| 80.25 | 5.0 | 9.5 | 6.0 | 56.2 | 24.0 | 1.20 | 156 | 11.6 |
| 101.50 | 5.6 | 9.1 | 6.3 | 1026.9 | 21.1 | 0.10 | #DIV/0! | 10.6 |
| 32.50 | 4.0 | 9.2 | 3.3 | 167.6 | 16.7 | 0.10 | 53 | 10.8 |
| 49.17 | 5.7 | 9.3 | 5.6 | 162.5 | 27.4 | 2.75 | 81 | 8.5 |
| 89.00 | 4.8 | 8.2 | 5.3 | 282.5 | 17.8 | 0.10 | #DIV/0! | 11.0 |
| 78.33 | 4.6 | 9.5 | 7.8 | 383.0 | 18.2 | 0.10 | 132 | 10.6 |
| 68.83 | 5.0 | 8.8 | 6.4 | 497.4 | 16.4 | 0.72 | 35 | 7.6 |
| 52.00 | 4.3 | 9.6 | 3.8 | 91.2 | 12.1 | 0.20 | #DIV/0! | 10.9 |
| 97.50 | 4.9 | 8.3 | 6.0 | 277.5 | 19.1 | 0.12 | #DIV/0! | 9.9 |
| 73.50 | 4.9 | 8.7 | 5.5 | 305.7 | 13.8 | 0.10 | #DIV/0! | 9.8 |
| 58.50 | 4.2 | 8.6 | 6.9 | 158.5 | 41.3 | 0.10 | #DIV/0! | 10.5 |
| 64.00 | 4.4 | 9.3 | 6.1 | 107.7 | 26.1 | 0.55 | 108 | 10.5 |
| 74.83 | 4.9 | 8.7 | 5.5 | 224.6 | 17.6 | 0.15 | 71 | 8.5 |
| 88.40 | 5.0 | 8.6 | 5.0 | 500.4 | 28.9 | 0.20 | 88 | 9.5 |
| 57.00 | 4.5 | 8.7 | 5.5 | 466.5 | 31.1 | 0.12 | 116 | 11.1 |
| 59.00 | 3.6 | 8.9 | 6.2 | 374.9 | 14.0 | 0.46 | 106 | 11.7 |
| 53.00 | 5.7 | 8.7 | 5.9 | 161.7 | 21.8 | 0.23 | 115 | 10.4 |
| 51.00 | 4.1 | 9.6 | 5.8 | 106.6 | 18.6 | 0.42 | #DIV/0! | 11.5 |
| 76.40 | 5.0 | 8.9 | 6.3 | 69.2 | 21.0 | 0.11 | #DIV/0! | 11.5 |
| 50.17 | 5.4 | 10.3 | 5.3 | 137.0 | 29.1 | 0.10 | #DIV/0! | 10.8 |
| 64.50 | 5.3 | 9.6 | 5.2 | 151.9 | #DIV/0! | 0.10 | 112 | 10.3 |
| 58.00 | 4.5 | 8.0 | 7.1 | 232.3 | 23.6 | 0.21 | 152 | 9.8 |
| 60.67 | 5.3 | 7.9 | 5.9 | 631.9 | 19.7 | 0.10 | #DIV/0! | 9.8 |
| 62.00 | 4.9 | 9.2 | 5.1 | 80.7 | 16.3 | 1.47 | #DIV/0! | 12.3 |
| 76.00 | 5.4 | 9.4 | 6.2 | 83.6 | 24.9 | 0.10 | #DIV/0! | 11.0 |
| 47.00 | 4.4 | 8.5 | 5.7 | 419.6 | 13.8 | 0.49 | #DIV/0! | 10.7 |
| 44.17 | 4.6 | 9.0 | 3.8 | 45.1 | 34.5 | 0.11 | 119 | 10.2 |
| 65.00 | 5.9 | 9.1 | 5.2 | 123.2 | 27.7 | 0.10 | #DIV/0! | 10.6 |
| 68.83 | 5.6 | 9.3 | 5.7 | 233.1 | 30.0 | 0.54 | #DIV/0! | 10.5 |
| 56.33 | 4.7 | 10.3 | 5.9 | 214.3 | 31.1 | 0.32 | #DIV/0! | 10.7 |
| 68.00 | 5.7 | 8.4 | 6.4 | 652.3 | 21.4 | 0.10 | 64 | 10.6 |
| 47.50 | 4.3 | 9.7 | 4.1 | 102.0 | 10.3 | 0.10 | 52 | 11.1 |

| | | | | | | | | |
|---|---|---|---|---|---|---|---|---|
| 45.33 | 5.4 | 8.9 | 6.0 | 120.1 | 24.9 | 0.10 | 88 | 10.5 |
| 65.67 | 4.9 | 9.1 | 7.4 | 313.0 | 20.5 | 0.34 | 185 | 10.6 |
| 76.17 | 5.4 | 8.8 | 5.3 | 112.2 | 35.2 | 0.24 | 154 | 12.0 |
| 47.38 | 4.5 | 9.4 | 4.1 | 213.0 | #DIV/0! | 0.10 | 90 | 11.2 |

| Dialysis session /week | Dialysis hour/session | HDP | Disposition | Death | Technique Failure |
|---|---|---|---|---|---|
| Unknown | Unknown | Unknown | 1 | 1 | 0 |
| 4 | 4 | 64 | 0 | 0 | 0 |
| 6 | 3 | 108 | 0 | 0 | 0 |
| 5 | 4 | 100 | 0 | 0 | 0 |
| 6 | 4 | 144 | 2 | 0 | 1 |
| 4 | 5.5 | 88 | 2 | 0 | 1 |
| 6 | 4 | 144 | 0 | 0 | 0 |
| 5 | 4 | 100 | 1 | 1 | 0 |
| 4 | 3 | 48 | 3 | 0 | 0 |
| 7 | 3 | 147 | 0 | 0 | 0 |
| 4 | 4 | 64 | 0 | 0 | 0 |
| 5 | 7 | 175 | 0 | 0 | 0 |
| 5 | 3 | 75 | 2 | 0 | 1 |
| 5 | 4 | 100 | 0 | 0 | 0 |
| 5 | 4 | 100 | 0 | 0 | 0 |
| 5 | 4 | 100 | 4 | 0 | 0 |
| 7 | 4 | 196 | 2 | 0 | 1 |
| 7 | 2 | 98 | 0 | 0 | 0 |
| 4 | 3 | 48 | 1 | 1 | 0 |
| 7 | 2 | 98 | 1 | 1 | 0 |
| 7 | 2 | 98 | 0 | 0 | 0 |
| 4 | 3.5 | 56 | 3 | 0 | 0 |
| 7 | 2 | 98 | 0 | 0 | 0 |
| 6 | 3 | 108 | 0 | 0 | 0 |
| 6 | 2 | 72 | 2 | 0 | 1 |
| 7 | 2.5 | 122.5 | 0 | 0 | 0 |
| 6 | 3 | 108 | 2 | 0 | 1 |
| 6 | 3 | 108 | 2 | 0 | 1 |
| 7 | 4 | 196 | 4 | 0 | 0 |
| 5 | 4.5 | 112.5 | 1 | 1 | 0 |
| 5 | 3 | 75 | 0 | 0 | 0 |
| 6 | 3.5 | 126 | 0 | 0 | 0 |
| 6 | 3 | 108 | 2 | 0 | 1 |
| 4 | 4 | 64 | 1 | 1 | 0 |
| 6 | 3 | 108 | 4 | 0 | 0 |
| 6 | 4 | 144 | 0 | 0 | 0 |

| | | | | | |
|---|---|---|---|---|---|
| 5 | 2.5 | 62.5 | 0 | 0 | 0 |
| 7 | 4 | 196 | 0 | 0 | 0 |
| 6 | 2 | 72 | 2 | 0 | 1 |
| 4 | 4 | 64 | 0 | 0 | 0 |
| 6 | 3.5 | 126 | 0 | 0 | 0 |
| 5 | 4 | 100 | 1 | 1 | 0 |
| 6 | 3 | 108 | 0 | 0 | 0 |
| 6 | 5 | 180 | 0 | 0 | 0 |
| 6 | 4 | 144 | 1 | 1 | 0 |
| 4 | 2.5 | 40 | 1 | 1 | 0 |
| 5 | 6 | 150 | 0 | 0 | 0 |
| 4 | 5 | 80 | 2 | 0 | 1 |
| 4 | 3 | 48 | 0 | 0 | 0 |
| 4 | 4 | 64 | 0 | 0 | 0 |
| 7 | 3 | 147 | 0 | 0 | 0 |
| 4 | 4 | 64 | 2 | 0 | 1 |
| 7 | 2.5 | 122.5 | 2 | 0 | 1 |
| 4 | 4 | 64 | 0 | 0 | 0 |
| 5 | 4 | 100 | 0 | 0 | 0 |
| 4 | 4.5 | 72 | 2 | 0 | 1 |
| 4 | 4 | 64 | 1 | 1 | 0 |
| 5 | 4 | 100 | 0 | 0 | 0 |
| 5 | 3 | 75 | 0 | 0 | 0 |
| 4 | 4 | 64 | 0 | 0 | 0 |
| 6 | 3 | 108 | 2 | 0 | 1 |
| 7 | 4 | 196 | 0 | 0 | 0 |
| 5 | 3 | 75 | 0 | 0 | 0 |
| 4 | 3 | 48 | 0 | 0 | 0 |
| 4 | 3 | 48 | 2 | 0 | 1 |
| 5 | 4.5 | 112.5 | 0 | 0 | 0 |
| 4 | 5 | 80 | 1 | 1 | 0 |
| 5 | 3 | 75 | 0 | 0 | 0 |
| 6 | 3 | 108 | 0 | 0 | 0 |
| 6 | 3 | 108 | 2 | 0 | 1 |
| 5 | 4 | 100 | 0 | 0 | 0 |
| 4 | 3 | 48 | 2 | 0 | 1 |
| 7 | 2.5 | 122.5 | 0 | 0 | 0 |

| | | | | | |
|---|---|---|---|---|---|
| 6 | 4 | 144 | 2 | 0 | 1 |
| 5 | 3 | 75 | 0 | 0 | 0 |
| 4 | 3.5 | 56 | 2 | 0 | 1 |
| 5 | 3 | 75 | 0 | 0 | 0 |

| Transplantation | Relocation | HHD initiation～endpoint（month） |
|---|---|---|
| 0 | 0 | 160 |
| 0 | 0 | 216 |
| 0 | 0 | 195 |
| 0 | 0 | 192 |
| 0 | 0 | 42 |
| 0 | 0 | 176 |
| 0 | 0 | 176 |
| 0 | 0 | 167 |
| 1 | 0 | 23 |
| 0 | 0 | 169 |
| 0 | 0 | 166 |
| 0 | 0 | 164 |
| 0 | 0 | 46 |
| 0 | 0 | 161 |
| 0 | 0 | 161 |
| 0 | 1 | 46 |
| 0 | 0 | 148 |
| 0 | 0 | 151 |
| 0 | 0 | 73 |
| 0 | 0 | 22 |
| 0 | 0 | 147 |
| 1 | 0 | 43 |
| 0 | 0 | 147 |
| 0 | 0 | 147 |
| 0 | 0 | 65 |
| 0 | 0 | 145 |
| 0 | 0 | 51 |
| 0 | 0 | 76 |
| 0 | 1 | 29 |
| 0 | 0 | 145 |
| 0 | 0 | 143 |
| 0 | 0 | 140 |
| 0 | 0 | 102 |
| 0 | 0 | 60 |
| 0 | 1 | 58 |
| 0 | 0 | 135 |

| | | |
|---|---|---|
| 0 | 0 | 135 |
| 0 | 0 | 134 |
| 0 | 0 | 133 |
| 0 | 0 | 134 |
| 0 | 0 | 133 |
| 0 | 0 | 74 |
| 0 | 0 | 131 |
| 0 | 0 | 128 |
| 0 | 0 | 59 |
| 0 | 0 | 47 |
| 0 | 0 | 126 |
| 0 | 0 | 104 |
| 0 | 0 | 126 |
| 0 | 0 | 125 |
| 0 | 0 | 125 |
| 0 | 0 | 26 |
| 0 | 0 | 120 |
| 0 | 0 | 123 |
| 0 | 0 | 122 |
| 0 | 0 | 129 |
| 0 | 0 | 91 |
| 0 | 0 | 116 |
| 0 | 0 | 116 |
| 0 | 0 | 116 |
| 0 | 0 | 35 |
| 0 | 0 | 114 |
| 0 | 0 | 113 |
| 0 | 0 | 112 |
| 0 | 0 | 62 |
| 0 | 0 | 108 |
| 0 | 0 | 59 |
| 0 | 0 | 102 |
| 0 | 0 | 102 |
| 0 | 0 | 72 |
| 0 | 0 | 98 |
| 0 | 0 | 64 |
| 0 | 0 | 93 |

| | | |
|---|---|---|
| 0 | 0 | 39 |
| 0 | 0 | 82 |
| 0 | 0 | 58 |
| 0 | 0 | 26 |
