# Supplementary material for: Long-term survival of patients receiving home hemodialysis with self-punctured arteriovenous access
Source: PLoS One. 2024 May 31;19(5):e0303055. doi: 10.1371/journal.pone.0303055 (PMC11142548; doi:10.1371/journal.pone.0303055)
Supplement: S2 File — (DOCX) [file pone.0303055.s002.docx]

在宅血液透析患者の長期予後の検討

研究計画書

　　　　　　　　　　　　　　　　　　　　　　　　　　研究責任者 　　　　　岡田浩一

＜研究機関名＞埼玉医科大学病院

　＜所属＞　　　　　　　腎臓内科

＜氏名＞　　　　　　　友利浩司

**１.　研究の目的**

　在宅血液透析患者の長期予後を明らかにすること。

**２.　研究の背景と意義**

在宅血液透析(Home Hemodialysis: HHD)は、患者宅で患者本人によって行われる透析モダリティである。HHDでは、患者のライフスタイルに合わせて、透析の頻度および時間を自由に設定できることから、頻回・長時間透析が可能となる。頻回透析は、患者QOLおよび生存率を改善させることが示されおり^1) 、2)^、Nishio-Lucarらは、頻回HHD患者と献腎移植患者の生存率は同等であり、HHDは移植困難な患者の治療オプションとして適していると報告している^2)^。したがって、腎移植の普及率が低く、長期透析を余儀なくされるわが国において、HHDは有望な透析モダリティと考えられる。しかし、2021年現在、わが国のHHD患者は748人であり、全透析患者のわずか0.2％にすぎない^3)^。このことからわが国においては、HHDの今後の普及が課題となっている。

一方でHHDを継続していくうえでは、バスキュラーアクセスに関連した合併症や感染症の増加^1)、 4)、5)^、残存腎機能の喪失^6)^、患者およびケアパートナーの負担の増加^7)^など、多くの問題点があることも指摘されている。Sehasaiらは、2007年から2009年にかけて米国内でHHDを導入した患者2,480人の予後を検証しており、1年間の離脱率は24.9%、死亡率は7.9%であり、糖尿病、喫煙・飲酒・薬剤使用、腎移植非適応、都市部居住が、治療中止のリスク因子であったと報告している^8)^。また、Paulyらは、カナダの夜間頻回透析患者を12年間追跡した結果、治療継続率は1年 95.2%、5年80.1%であり、年齢と糖尿病が治療中止の予測因子であったと報告している^9)^。さらに英国のHHD患者166人を8年間4,528人月観察したJayantiらの報告では、治療継続率は、1年90.2％、5年81.5%であり、糖尿病と心不全が治療失敗の予測因子であることが示されている^10)^。このようにHHDの継続率は、研究の対象となった国、コホートによって大きく異なるが、これらのエビデンスは、全て海外からの報告に基づいており、人種、医療経済および腎不全医療の実情が大きく異なるわが国において、そのまま外挿することは困難である。わが国からのエビデンスの発信が求められている。

そこで本研究では、埼玉医科大学病院Saitama medical university HHD programでHHDを導入した患者を対象として、HHD患者の長期予後を検証することを目的とする。具体的には、治療継続率とその予測因子、HHD離脱原因の詳細を明らかにする。本研究の結果は、わが国におけるHHDの普及と長期継続という重要な課題に対して有意義なエビデンスを提供するものと考えられる。

参考文献）

1. Chertow GM, Levin NW, Beck GJ, Daugirdas JT, Eggers PW, Kliger AS, Larive B, Rocco MV, Greene T; Frequent Hemodialysis Network (FHN) Trials Group. Long-Term Effects of Frequent In-Center Hemodialysis. J Am Soc Nephrol. 2016 Jun;27(6):1830-6.
2. Nishio-Lucar AG, Bose S, Lyons G, Awuah KT, Ma JZ, Lockridge RS Jr. Intensive Home Hemodialysis Survival Comparable to Deceased Donor Kidney Transplantation. Kidney Int Rep. 2020
3. 日本透析医学会統計調査委員会. わが国の慢性透析療法の現況. 透析会誌 55(12). 665-723, 2022
4. Suri RS, Larive B, Sherer S, Eggers P, Gassman J, James SH, Lindsay RM, Lockridge RS, Ornt DB, Rocco MV, Ting GO, Kliger AS; Frequent Hemodialysis Network Trial Group. Risk of vascular access complications with frequent hemodialysis. J Am Soc Nephrol. 2013
5. Weinhandl ED, Nieman KM, Gilbertson DT, Collins AJ. Hospitalization in daily home hemodialysis and matched thrice-weekly in-center hemodialysis patients. Am J Kidney Dis. 2015 Jan;65(1):98-108.
6. Daugirdas JT, Greene T, Rocco MV, Kaysen GA, Depner TA, Levin NW, Chertow GM, Ornt DB, Raimann JG, Larive B, Kliger AS; FHN Trial Group. Effect of frequent hemodialysis on residual kidney function. Kidney Int. 2013 May;83(5):949-58.
7. Suri RS, Larive B, Garg AX, Hall YN, Pierratos A, Chertow GM, Gorodetskeya I, Kliger AS; FHN Study Group. Burden on caregivers as perceived by hemodialysis patients in the Frequent Hemodialysis Network (FHN) trials. Nephrol Dial Transplant. 2011 Jul;26(7):2316-22.
8. Seshasai RK, Mitra N, Chaknos CM, Li J, Wirtalla C, Negoianu D, Glickman JD, Dember LM. Factors Associated With Discontinuation of Home Hemodialysis. Am J Kidney Dis. 2016 Apr;67(4):629-37.
9. Pauly RP, Maximova K, Coppens J, Asad RA, Pierratos A, Komenda P, Copland M, Nesrallah GE, Levin A, Chery A, Chan CT; CAN-SLEEP Collaborative Group. Patient and technique survival among a Canadian multicenter nocturnal home hemodialysis cohort. Clin J Am Soc Nephrol. 2010 Oct;5(10):1815-20.
10. Jayanti A, Nikam M, Ebah L, Dutton G, Morris J, Mitra S. Technique survival in home haemodialysis: a composite success rate and its risk predictors in a prospective longitudinal cohort from a tertiary renal network programme. Nephrol Dial Transplant. 2013

**３．研究の実施体制・組織**

１）研究責任者

　　所属　腎臓内科　　（役職　部長）　　　氏名　岡田浩一

２）研究実施者：別紙記載

３）研究事務局及び担当者（該当する場合）：該当なし

４）上記以外のデータセンターなどの外部機関（該当する場合）：該当なし

**４.　研究の方法と期間**

１）研究対象者の定義：埼玉医科大学病院で在宅血液透析を導入した全患者とし、データ欠損者と3か月以内の早期離脱者は除外する。

２）目標症例数

　　症例数　　（77）例

３）研究の期間

調査対象期間：2001年1月1日　～　2021年12月31日

研究期間：承認日　～　2024年12月31日

４）調査項目/検査項目

　患者背景（性別、HHD導入時年齢、末期腎不全の原疾患、併存疾患、HHD導入前の腎代替療法歴と種類、HHD導入時の身長・体重、血液検査結果）、HHD治療の内容（透析時間、週当たりの透析回数、バスキュラーアクセスの種類、穿刺法）、および患者転帰（死亡、転院、移植、technique failure : TF）を調査する。

技術的失敗（トレーニング失敗、死亡、移植以外の理由で在宅HD療法を中止したものと定義）および死亡率。

５）統計処理を行う方法

HHD導入時の患者背景について、標準的な記述統計を使用して集計し要約する。主要評価項目はHHD治療継続率とし、全死亡とTFの複合を主要イベントと定義し、カプランマイヤー法でイベントフリー生存率を推定する。治療継続に関連する因子を特定するため、cox比例ハザードモデルを用いる。まず、各変数と治療継続率との関連を単変量解析で調べ、次に有意な変数を含めた多変量解析を行い、治療継続に関連する独立した因子を特定する。

６）その他

**５.　研究に関する情報公開について**

**（後方視的観察研究の場合必須ではないので、該当する場合のみ）**

研究開始時のデータベースへの登録：

データベースの名称：（UMIN臨床試験登録システム）

登録番号：（R000057952）

**６．試料・情報の保管について**

　調査対象データは施設の電子カルテ内に適切に保管されている。担当医師は基準に該当する症例を選択して登録し、研究に必要なデータを取得、調査票を作成する。その際、ID

や氏名など個人を特定出来る情報は削除され、本研究に特有の症例No.が付与される。研究用に加工した調査情報はログインパスワードが必要なパソコン内で保管する。同パソコンは施錠可能な医局内に設置されており、夜間休日は同室の入り口は施錠される。特定の個人を識別できない様に加工する際の症例No.と各施設のIDとの対応表は作成しない（再調査は想定していない）。調査票は研究終了5 年後に完全に廃棄される。情報については共同研究終了後から10年間適切に保管するが、本研究のためのみに使用する。

**７．個人情報保護の方法**

当院単独研究であり、情報（データ）の解析等もすべて当院で実施するため、研究実施中に被験者の氏名、ID、生年月日などの個人情報が、外部に出ることはない。公表時にも被験者の個人情報保護については十分に配慮する。

**８．インフォームドコンセント**

後ろ向き研究のため、被験者からインフォームドコンセントは受けないが、研究の情報を公開し、研究対象者が拒否できる機会を保障する。

　情報公開する場所

　埼玉医科大学病院ＩＲＢホームページ

　 URL: 　https://saitama-med.bvits.com/rinri/publish.aspx?BOARD_ID=1

**９．研究に関する被験者からの相談等の対応**

以下の連絡先を相談窓口とする。

連絡先：埼玉医科大学病院　（　腎臓内科　）

電話番号：（　049-276-1611　）

**１０．費用に関する事項**

１）研究の資金源

２）研究に係る利益相反について

この研究を実施するにあたり、当院における研究者の利益相反については、埼玉医科大学病院COI管理委員会に申告するなどして適正に管理されている。

**１１．病院長への報告に関する事項**

1. 研究の実施の許可：

研究責任者は、研究の実施に先立ち、本研究計画書について病院ＩＲＢの承認及び病院長の許可を得ていることを確認する。

1. 研究計画内容の変更：

研究責任者は、研究計画書内容に変更点が生じた場合は、速やかに変更申請し、病院ＩＲＢの承認を得て、病院長の許可を得る。

1. 実施状況報告：

研究責任者は、少なくとも年に１回以上の頻度で、研究の実施状況を病院ＩＲＢ及び病院長に報告する。

1. 研究終了時：

研究責任者は、研究が終了したら速やかに病院ＩＲＢと病院長に報告する。

**１２．研究結果の公表**

学術論文として公表する。

**１３．知的財産権について**

本研究の成果により、知的財産権が生じる可能性がある。その権利は埼玉医科大学に属し、被験者に知的財産権は属さない。

＜別紙＞　研究組織　一覧

１）研究実施者

| 氏名 | 所属（役職） |
| --- | --- |
|  |  |
| 友利浩司 | 腎臓内科（講師） |
| 井上　勉 | 腎臓内科（教授） |
| 渡辺祐輔 | 腎臓内科（准教授） |
| 天野博明 | 腎臓内科（助教） |
| 伊藤悠人 | 腎臓内科（助教） |
| 近藤立雄 | 腎臓内科（助教） |
| 村杉浩 | 臨床工学部（主任） |
| 大橋直人 | 臨床工学部 |
| 杉山正夫 | 臨床工学部 |

２）研究事務局及び担当者（該当する場合）

　該当なし

３）データセンターなどの外部機関　（該当する場合）

　該当なし
