# Supplementary material for: Long-term survival of patients receiving home hemodialysis with self-punctured arteriovenous access
Source: PLoS One. 2024 May 31;19(5):e0303055. doi: 10.1371/journal.pone.0303055 (PMC11142548; doi:10.1371/journal.pone.0303055)
Supplement: S3 File — (DOCX) [file pone.0303055.s003.docx]

Long-term prognosis of intensive home hemodialysis patients

Study protocol

Principal Investigator: Hirokazu Okada

Institution: Saitama Medical University Hospital

　Department: Nephrology

**1. Objective of the study**

　To determine the long-term prognosis of home hemodialysis patients.

**2. Background and Significance of the Study**

Home Hemodialysis (HHD) is a dialysis modality performed by the patient in the patient's home. HHD allows for frequent and prolonged dialysis, with the freedom to set the frequency and duration of dialysis to suit the patient's lifestyle. Frequent dialysis has been shown to improve patient quality of life and survival^1) 、2)^ , and Nishio-Lucar et al. reported that the survival rates of frequent HHD patients and cadaveric kidney transplant patients are comparable, making HHD a suitable treatment option for difficult-to-transplant patients . ^2)^Therefore, HHD is considered a promising dialysis modality in Japan, where kidney transplantation is not widely available and patients are forced to undergo long-term dialysis. However, as of 2021, there are 748 HHD patients in our country, which is only 0.2% of all dialysis patients.^3)^ This makes the future spread of HHD a challenge in our country.

On the other hand, there are many problems associated with continuing HHD, including increased complications and infections related to vascular access^1)、 4)、5)^ , loss of residual renal function^6)^ , and increased burden on patients and care partners^7)^ . Sehasai et al. reviewed the outcomes of 2,480 patients with HHD in the United States between 2007 and 2009. They reported a one-year withdrawal rate of 24.9% and a mortality rate of 7.9%, with diabetes, smoking, alcohol and drug use, non-eligibility for renal transplantation, and urban residence as risk factors for treatment discontinuation.^8)^ Pauly et al. also reported that in a 12-year follow-up of Canadian patients on nocturnal frequent dialysis, treatment retention was 95.2% at 1 year and 80.1% at 5 years, with age and diabetes being predictors of treatment discontinuation.^9)^ Furthermore, Jayanti et al. in an 8-year, 4,528 person-month observation of 166 HHD patients in the UK reported treatment continuation rates of 90.2% at 1 year and 81.5% at 5 years, indicating that diabetes and heart failure were predictors of treatment failure.^10)^ Thus, HHD persistence rates vary widely by country and cohort studied, but all of this evidence is based on reports from overseas and is difficult to extrapolate directly to Japan, where race, health care economics, and the reality of renal failure care are very different. The dissemination of evidence from Japan is needed.

Therefore, the purpose of this study is to examine the long-term prognosis of HHD patients who were introduced to HHD at the Saitama Medical University HHD program. Specifically, we will clarify details of treatment retention rates, its predictors, and causes of HHD withdrawal. The results of this study will provide meaningful evidence for the important issue of HHD diffusion and long-term continuation in Japan.

References

1. Chertow GM, Levin NW, Beck GJ, Daugirdas JT, Eggers PW, Kliger AS, Larive B, Rocco MV, Greene T; Frequent Hemodialysis Network (FHN) Trials Group: Long- Term Effects of Frequent In-Center Hemodialysis. J Am Soc Nephrol. 2016 Jun;27(6):1830-6.
2. Nishio-Lucar AG, Bose S, Lyons G, Awuah KT, Ma JZ, Lockridge RS Jr. Intensive Home Hemodialysis Survival Comparable to Deceased Donor Kidney Kidney Int Rep. 2020
3. Committee on Statistics and Surveys, Japanese Society for Dialysis Therapy. Current status of chronic dialysis therapy in Japan. Journal of Dialysis Society of Japan 55(12). 665-723, 2022
4. Suri RS, Larive B, Sherer S, Eggers P, Gassman J, James SH, Lindsay RM, Lockridge RS, Ornt DB, Rocco MV, Ting GO, Kliger AS; Frequent Hemodialysis Network Trial Group. risk of vascular access complications with frequent hemodialysis.
5. Weinhandl ED, Nieman KM, Gilbertson DT, Collins AJ. hospitalization in daily home hemodialysis and matched thrice-weekly in-center hemodialysis patients. am J Kidney Dis. 2015 Jan;65(1):98-108.
6. Daugirdas JT, Greene T, Rocco MV, Kaysen GA, Depner TA, Levin NW, Chertow GM, Ornt DB, Raimann JG, Larive B, Kliger AS; FHN Trial Group. Effect of frequent Kidney Int. 2013 May;83(5):949-58.
7. Suri RS, Larive B, Garg AX, Hall YN, Pierratos A, Chertow GM, Gorodetskeya I, Kliger AS; FHN Study Group. Burden on caregivers as perceived by hemodialysis patients in the Frequent Hemodialysis Network (FHN) trials. Nephrol Dial Transplant. 2011 Jul;26(7):2316-22.
8. Seshasai RK, Mitra N, Chaknos CM, Li J, Wirtalla C, Negoianu D, Glickman JD, Dember LM. Factors Associated With Discontinuation of Home Hemodialysis. Am J Kidney Dis. 2016 Apr;67(4):629-37.
9. Pauly RP, Maximova K, Coppens J, Asad RA, Pierratos A, Komenda P, Copland M, Nesrallah GE, Levin A, Chery A, Chan CT; CAN-SLEEP Collaborative Group. Patient and technique survival among a Canadian multicenter nocturnal home hemodialysis cohort. Clin J Am Soc Nephrol. 2010 Oct;5(10):1815-20.
10. Jayanti A, Nikam M, Ebah L, Dutton G, Morris J, Mitra S. Technique survival in home haemodialysis: a composite success rate and its risk predictors in a Nephrol Dial Transplant. 2013.

**3. Organization of the study**

(1) Principal investigator

　　Department: Nephrology

Name: Hirokazu Okada (Professor)

(2) Investigator: Listed separately

(3) Research office and contact person (if applicable): Not applicable

(4) External organizations such as data centers other than the above (if applicable): Not applicable

**4. Method and duration of the study**

1) Definition of study subjects: All patients who were introduced to home hemodialysis at Saitama Medical University Hospital, excluding those with missing data and those who withdrew early within 3 months.

(2) Target number of cases

　　Number of cases (77) cases

(3) Duration of the study

Survey period: January 1, 2001 - December 31, 2021

Research period: Date of approval - December 31, 2024

(4) Survey Items/Inspection Items

Patient background (gender, age at HHD induction, primary end-stage renal disease, comorbidities, history and type of renal replacement therapy prior to HHD induction, height and weight at HHD induction, blood test results), HHD treatment (dialysis time, number of dialyses per week, type of vascular access, puncture technique), and patient outcome (death, relocation, transplant, technique failure : TF) will be investigated.

TF is defined as discontinuation of HHD for more than 60 days for reasons other than death, transfer, or transplantation.

(5) Method of statistical processing

Patient background at HHD induction will be tabulated and summarized using standard descriptive statistics. The primary endpoint will be HHD treatment retention, and the composite of all-cause mortality and TF will be defined as the primary event, and event-free survival will be estimated using the Kaplan-Meier method. A cox proportional hazards model will be used to identify factors associated with treatment retention. First, the association between each variable and treatment retention rate will be examined in a univariate analysis, followed by a multivariate analysis including significant variables to identify independent factors associated with treatment retention.

(6) Others: Not specified

**5. Disclosure of information on research**

**(Not required for retrospective observational studies, only if applicable)**

Registration in the database at the beginning of the study:

Database name: (UMIN Clinical Trial Registration System)

Registration number: (R000057952)

**6. Storage of Samples and Information**

　The data to be studied are appropriately stored in the facility's electronic medical record. The physician in charge selects and registers cases that meet the criteria, obtains the data necessary for the study, and prepares the survey form. At that time, an ID

All personally identifiable information (e.g., names, etc.) will be deleted, and a case number specific to this study will be assigned. The survey information processed for research purposes will be stored in a computer that requires a login password. The computer is located in a lockable doctor's office, and the entrance to the office is locked at night and on holidays. When processing the data so that specific individuals cannot be identified, no correspondence sheet between the case No. and the ID of each facility will be created (re-survey is not anticipated). The questionnaires will be completely destroyed after 5 years from the end of the study. Information will be appropriately stored for 10 years after the end of the collaborative study, but will be used only for this study.

**7. privacy protection methods**

Since the research is conducted solely at this hospital and all information (data) analysis, etc. will be conducted at this hospital, no personal information such as subjects' names, IDs, or dates of birth will be disclosed outside the hospital during the research. We will also give due consideration to the protection of the subjects' personal information at the time of publication.

**8. informed consent**

Because it is a backward-looking study, informed consent will not be obtained from the subjects, but information about the study will be made publicly available and the opportunity for the subjects to refuse will be guaranteed.

　Information disclosure location: Saitama Medical School Hospital IRB Website

　 URL: https://saitama-med.bvits.com/rinri/publish.aspx?BOARD_ID=1

**9. Response to queries and concerns from participants**

Contact: Saitama Medical University Hospital (Nephrology)

Phone number: (049-276-1611)

**10. Financial matters**

1) Sources of funding for research:

2) Conflicts of Interest Related to Research

In conducting this research, conflicts of interest of researchers in our hospital are properly managed by declaring them to the COI Management Committee of Saitama Medical University Hospital.

**11. Reporting to the hospital director**

1. Permission to conduct research:

The principal investigator confirms that approval has been obtained from the hospital IRB and permission from the hospital director before initiating the research.

1. Amendments to the study protocol:

Should there be any changes to the study protocol, the principal

investigator will promptly apply for a modification, obtain the hospital IRB's approval, and then secure permission from the hospital director.

1. Reporting on the research status:

The principal investigator will report the research's status to both the hospital director and the hospital IRB at least once a year.

４）Research completion:

The principal investigator will report to both the hospital director and the hospital IRB as soon as the research is completed.

**12．Publication of research results**

Results will be published in a journal.

**13．Intellectual property rights**

The outcome of this research may result in intellectual property rights. Any such rights will belong to Saitama Medical University, and the participants will have no claims to them.

＜List of research organizations

(1) Research implementer

| identity | Affiliation (Position) |
| --- | --- |
|  |  |
| Koji Tomori | Nephrology (Lecturer) |
| Tsutomu Inoue | Nephrology (Professor) |
| Yusuke Watanabe | Nephrology (Associate Professor) |
| Hiroaki Amano | Nephrology (Assistant Professor) |
| Yuto Ito | Nephrology (Assistant Professor) |
| Tatsuo Kondo | Nephrology (Assistant Professor) |
| Hiroshi Murasugi | Clinical Engineering (Chief) |
| Naoto Ohashi | Clinical Engineering Department |
| Masao Sugiyama | Clinical Engineering Department |
