# Supplementary material for: Long-term survival of patients receiving home hemodialysis with self-punctured arteriovenous access
Source: PLoS One. 2024 May 31;19(5):e0303055. doi: 10.1371/journal.pone.0303055 (PMC11142548; doi:10.1371/journal.pone.0303055)
Supplement: Attachment — Submitted filename: Response_to_Reviewer.docx [file pone.0303055.s004.docx]

We have enclosed our revised manuscript entitled “Long-term survival of patients receiving home hemodialysis with self-punctured arteriovenous access” for publication as an original article in PLOS ONE. Our point-by-point responses to the reviewers’ comments are as follows:

To Reviewer 1

1．*The lack of a control group to compare the results with. For example, a control group could consist of in-center hemodialysis patients from the same dialysis facility, matched for age and comorbidities, over a relevant follow-up period.*

Response:

Thank you for your meaningful comment. As you have pointed out, our study did not include a control group, which we acknowledge as a limitation of our research. Similarly, previous studies investigating technique survival did not set a control group [1-6] (Table 5). The primary aim of our observational study was to explore the long-term treatment survival among patients receiving HHD with self-punctured arteriovenous access, considering a composite outcome that includes both death and technique failure. We did not include in-center hemodialysis patients as a control group because technique failure, as defined in our study, does not occur in in-center hemodialysis settings. We have added this description to the Discussion section as a limitation of our study (P26, L307–313).

1) Pauly RP, Maximova K, Coppens J, Asad RA, Pierratos A, Komenda P, et al. Patient and technique survival among a Canadian multicenter nocturnal home hemodialysis cohort. Clin J Am Soc Nephrol. 2010;5: 1815-1820. doi: [10.2215/CJN.00300110](https://doi.org/10.2215/cjn.00300110).
2) Jun M, Jardine MJ, Gray N, Masterson R, Kerr PG, Agar JW, et al. Outcomes of extended-hours hemodialysis performed predominantly at home. Am J Kidney Dis. 2013;61: 247-253. doi: [10.1053/j.ajkd.2012.08.032](https://doi.org/10.1053/j.ajkd.2012.08.032).
3) Jayanti A, Nikam M, Ebah L, Dutton G, Morris J, Mitra S. Technique survival in home haemodialysis: a composite success rate and its risk predictors in a prospective longitudinal cohort from a tertiary renal network programme. Nephrol Dial Transplant. 2013;28: 2612-2620. doi: [10.1093/ndt/gft294](https://doi.org/10.1093/ndt/gft294).
4) Seshasai RK, Mitra N, Chaknos CM, Li J, Wirtalla C, Negoianu D, et al. Factors associated with discontinuation of home hemodialysis. Am J Kidney Dis. 2016;67: 629-637. doi: [10.1053/j.ajkd.2015.11.003](https://doi.org/10.1053/j.ajkd.2015.11.003).
5) Pauly RP, Rosychuk RJ, Usman I, Reintjes F, Muneer M, Chan CT, et al. Technique failure in a multicenter Canadian home hemodialysis cohort. Am J Kidney Dis. 2019;73: 230-239. doi: [10.1053/j.ajkd.2018.08.016](https://doi.org/10.1053/j.ajkd.2018.08.016).
6) Paterson B, Fox DE, Lee CH, Riehl-Tonn V, Qirzaji E, Quinn R, et al. Understanding home hemodialysis patient attrition: a cohort study. Can J Kidney Health Dis. 2021;8: 20543581211022195. doi: [10.1177/20543581211022195](https://doi.org/10.1177/20543581211022195).

*2. The study included home hemodialysis patients as one group, regardless of their previous kidney replacement therapy. This could potentially affect the results, as patients with prior kidney replacement therapies, especially in-center hemodialysis, or kidney transplantation, may have been exposed to additional factors compared to patients who started home hemodialysis directly.*

Response:

We agree with the reviewer's comment. Recognizing the importance of previous KRT, we conducted additional analysis, stratifying patients based on their pre-HHD modality. We categorized patients into three groups: those who directly initiated HHD, transitioned from in-center HD, and switched from PD to HHD. Due to the presence of only one patient who had transitioned from kidney transplantation, we excluded this patient from the analysis.

We found no significant differences in treatment survival between the remaining two groups. This suggests that, within our study population, the previous KRT modality did not significantly affect the long-term outcomes of patients who transitioned to HHD. We have added this description to the Discussion section (P24, L252–L257) and detailed the previous KRT modalities in Table 1. In addition, we have corrected errors related to the frequencies of pre-HHD modalities as initially reported in the body of the manuscript (P8, L136).

1) Morin C, Gionest I, Laurin LP, Goupil R, Nadeau-Fredette AC. Risk of hospitalization, technique failure, and death with increased training duration in 3-days-a-week home hemodialysis. Hemodial Int. 2021;25(4):457-464. doi:10.1111/hdi.12956

3．*Although patient education before starting home hemodialysis is considered an important issue, there is no mention of the education practices in the dialysis facility. How long was the education provided? What were the means of education?*

Response:

Thank you for highlighting the importance of patient education before initiating HHD. At our facility, patient education is provided by specialized staff three times a week, with a median duration of 102 days. We have added these data to Table 1 and Results (P8–9, L138–140).

As you pointed out, reports suggest that the training duration may be associated with technique survival [1]. In response to this, we included the training duration as a covariate in the additional analysis. However, this analysis did not show a significant association (HR 1.003, 95% CI 0.99–1.01, P=0.15), and we have discussed these results in the manuscript (P22–23, L248–251).

To Reviewer 2

*In this retrospective study, the authors describe a very favorable technical survival (defined as death or treatment failure) in their cohort of patients. Although this was a monocentric study with a relatively small cohort, it was followed over the long term for 19 years, one of the longest reported in the literature. The cohort is well described, attrition bias is low, and the statistical tests used seemed relevant and correctly conducted. As the authors point out, the transposability of the results may be limited, as the hemodialysis regimen varies with the country and health system. The system used is specific to their center using high dialysate flow hemodialysis rather than low flow. Another special feature is using not only frequent HD, but also relatively long session times .As the authors point out too, the center effect is important in home hemodialysis, and results depend on the center's experience, the type of patients treated and the resources deployed. However, the very favourable long-term experience with a low drop-out rate is interesting to consider, and the risk factors of age and diabetes on the one hand, and the quality of the vascular approach on the other, are well characterized.*

Response:

We greatly appreciate your thoughtful analysis and positive remarks on our study's findings regarding the technical survival of our patient cohort. We recognize the lack of HHD-specific dialysis machines in Japan, leading to the use of standard in-center machines for HHD. This results in differences in dialysate flow rates and other technique aspects compared to other dialysis practices and healthcare systems globally, which might limit the generalizability of our findings. We have added this description to the Discussion section as a limitation of our study (P26–27, L302–306) .

We thank you and the reviewers for their kind suggestions to improve the quality of our manuscript. We hope that our revised manuscript is suitable for publication as an original article in PLOS ONE.

Sincerely,

Hirokazu Okada, MD, PhD

Department of Nephrology, Faculty of Medicine, Saitama Medical University

38 Morohongo, Moroyama-cho, Iruma-gun, Saitama 350-0495, Japan.

TEL & FAX: +81-49-276-1111, E-Mail: hirookda@saitama-med.ac.jp
